# The Pennsylvania Hospital for the Insane

**Published:** 1850-07-01

**Authors:** 


### Art. IV.—THE PENNSYLVANIA HOSPITAL FOR THE INSANE.

In one of the early numbers of this Journal, we entered at some length into a history of the *Bloomingdale Asylum, New York.* We now propose (having the data before us) giving the history and selections from the Reports of another large American asylum, and we feel more pleasure in doing so, having heard very favourable accounts of the institution from all those who have had opportunities of personally inspecting it. The Reports of this asylum, from the first year of its being opened until 1850, have been kindly forwarded to us by Dr. Kirkbride, the resident physician, and from them we glean the following facts. It appears that the Pennsylvania Hospital is the oldest institution in America in which regular provision was made for the treatment of insanity, and it is said that within its walls were first promulgated many of the enlightened views from which so many important results have followed. Again: It appears that the fame of its early and eminent physicians and surgeons attracted crowds of students to its wards, and the influence of the practical instruction there obtained, has been felt in nearly every section of the Union.

"The first patient was admitted into the Pennsylvania Hospital on the 11th of the Second month, 1752, and between that time and the 20th of the Third month, 1841—when persons afflicted with mental derangement ceased to be received into the hospital in the city—*thirty-eight thousand four hundred* patients had received the benefits of its care. Of these, *four thousand three hundred and sixty-six* were insane, *one thousand four hundred and ninety-three* of whom were restored to their families perfectly cured—*nine hundred and thirteen* were discharged improved—*nine hundred and ninety-five* were removed by their friends without material improvement—*two hundred and forty-six* eloped*—*six hundred and ten* died—*ninety-three* were transferred to the new hospital, and *sixteen* were retained in the city, waiting the completion of the detached buildings. Of the 38,400 patients mentioned above, no less than *twenty thousand eight hundred and five* were received and supported without charge of any kind."—*First Report*, pp. 6, 7.

"All classes of insane persons, without regard to the duration of the disease, or of its curability, are admitted into this institution, upon securing the payment of a reasonable rate of board, by the obligation of some responsible resident of the city or county of Philadelphia."—p. 19.

A certain number of patients are admitted gratuitously. There are generally in the hospital from 100 to 130 free patients. There is no distinction made in the accommodation of the two classes of patients. The asylum is under the direction of a board of twelve managers, who are elected by the subscribers. To this board is intrusted the regulation of the funds, the election of the physicians: they are also required to visit the asylum once a week. The officers of the hospital consist of—

"*A physician,* who resides upon the premises, and to whom is confided the general superintendence of the establishment; the sole direction of the medical, moral, and dietetic treatment of the patients, and the selection or approval of all persons employed in their care.

"*An assistant physician,* living in the hospital, who prepares and dispenses all medicines prescribed for the patients, devotes himself to their care, sees that all directions respecting them are faithfully carried out, and that the attendants and others employed in the wards fail not in the performance of their duties.

"*A steward,* who takes care that the buildings and grounds are kept in good order, makes all the purchases for the house, receives all monies due to the institution for board, &c.; makes engagements with those employed, pays them for their services, and settles all accounts against the hospital.

"*A matron,* who has the general charge of the domestic economy of the house, the cooking and distribution of the food, and of the female domestics, and attends specially to the comfort of the female patients.

"In the wings, we have the assistance of the following persons,—

"*Supervisors,* one for each sex, whose duty it is to pass their time

* Mostly in the early days of the hospital, before the square was permanently closed.

among the patients in the different wards and pleasure-grounds, to endeavour to interest, employ, and amuse them in every way in their power, and to see that all the rules for the attendants in their intercourse with the patients are rigorously observed. Before retiring at night, the supervisors furnish the physician with a written report of whatever has come under their observation during the day.

"*Attendants*, who have the immediate care of the patients, sleep in the same divisions of the house, attend them in the dining-rooms, accompany them in their walks, rides, or amusements; assist them when engaged in manual labour, and take the entire charge of the halls, chambers, and clothing of the patients, as may be directed by the physician.

"At present, we have rather less than one attendant for every six patients.

"To these situations we endeavour to appoint none but those who are strictly temperate, moral, and of good intelligence. To perform *perfectly* the duties of attendant, requires such a variety of qualifications—such peculiar mental and physical endowments, as are not often combined in the same individual—that in all our engagements, it is understood that no one is expected to remain in the station who is found deficient in the qualities we deem essential to its proper performance."—*First Report*, pp. 21, 22.

In the Second Report we find the following observations:—

"*On the Importance of Early Treatment.*—Not a month elapses that we do not have to regret, that some individual is placed under our care after the best period for restorative treatment has passed. The general proposition, that truly recent cases of insanity are commonly very curable, and that chronic ones are only occasionally so, may be considered as fully established, and ought at this day to be everywhere understood.

"The feeling which prompts to retaining a friend under the care of kindred or acquaintances, is of the most natural and commendable kind,—but insanity derives one of its painful characteristics from the fact, now well ascertained, that in a large majority of cases, it can be managed with success only among strangers, and generally in institutions where extensive provision has been made for a liberal and enlightened treatment of this class of diseases.

"In private families, too, the care of recent cases of violent mania, to say nothing of the expense, is of the most painful character, and harrowing to the feelings of relatives in the highest degree. In the alarm attendant upon such attacks, with few persons capable of rendering assistance, and they ignorant of the proper means to be resorted to, restraint of the most violent and improper kind is often imposed, engendering feelings in the patients towards their friends, that last long after they have ceased to be insane. Even during the last year, we have received four patients who had been chained at their own homes, and who were capable of feeling, and did not fail to express their sense of the degradation. No one of these ever required restraining apparatus of any kind after entering this hospital. These facts are not mentioned to censure those who employed this form of restraint; situated as they were, it was difficult to do otherwise, but it is certainly a strong argument for placing the insane where such means are never used.

"Another difficulty, frequently arising from patients not being placed under treatment soon after the accession of the disease, is, that in chronic cases, their friends become disappointed if improvement is not promptly visible, and remove them in the midst of a course of treatment, before it is possible to predict whether it will be successful or not.

"Although some few recent cases of insanity do recover in a very few weeks, in the majority, even of these, a much longer time is required; and in the chronic, several months, often a whole year, is necessary to give trial to the means that may be employed. Less than the period last named should never be thought sufficient to destroy hopes of a recovery; and after a much longer time, we occasionally have the satisfaction of seeing patients perfectly restored. There are really some cases, in which it would be better for all parties to avoid the excitement and expense of removing them from home, unless there was a determination to persevere for a reasonable time in a trial of remedies. The board of managers require payment to be made for thirteen weeks' board of every *uncured* patient removed before that period, and contrary to the advice and consent of the superintending physician,—not with the wish to indicate this as sufficient for a trial of remedies, but only because, whenever a patient is removed without adequate reason, the payment of board for a less period does not compensate the institution for the inconvenience and disadvantage attending such a course, and for the expense to which it has been subjected.

"Although the importance of these views is now generally conceded by professional men, it is proper they should be believed by the friends of patients. No one should ever be placed in any insane hospital, until his friends are thoroughly satisfied of the importance and necessity of the measure; and then, only in an institution in whose organization and management they have entire confidence, and to the discretion and judgment of whose officers they are willing to submit the direction of the case.

"This kind of confidence, and the influence of friends springing from it, is often of great importance, in enabling the officers of an institution to carry out many details in both medical and moral treatment, and in preventing premature removals. Although in a few instances the want of it has disappointed our hopes, I am happy to be able to say, that, generally, we have been most faithfully seconded in our endeavours by the friends of patients.

"A strictly charitable institution like the Pennsylvania Hospital, depending for its principal support upon the contributions of the benevolent—its only object to relieve suffering humanity, and its income all expended for this purpose, and for this alone,—has the strongest reason for believing this confidence possessed by all who ask it to receive a patient within its walls. We have occasionally found this trait in the character of the institution highly gratifying to patients, and have seen the entire removal of fears of arbitrary or interested detention, by the discovery that no one connected with its management or direction had a pecuniary interest in the detention of patients; and that, however large the sum paid for their accommodations, the surplus over actual cost only went to impart some of the same comforts and advantages to those with whom poverty was added to disease.

"The economy of subjecting cases of mental derangement to proper treatment, immediately upon the occurrence of an attack, has not been generally understood, or no State would have neglected to make adequate provision for the early care of all who were thus afflicted. There can be no question but that every community, not having within itself the proper means, would save largely by sending their recent cases to some well-conducted insane hospital, and retaining them there as long as there was a prospect for their restoration. If this was done, a large proportion of them would, in a few months, be restored to society, instead of continuing, as is now too apt to be the case, a charge to their friends or the public during the remainder of their lives."—*Second Report*, pp. 22-5.

The following judicious observations are made relative to the visits of friends and others to patients in confinement:—

"When it has been decided that it is proper to place a patient in an insane hospital, the propriety of visits from acquaintances must always be left to the judgment of those to whom the management of the case has been entrusted. The welfare of the patient often demands that they should be completely interdicted, and a neglect of this precaution occasionally produces mortification and disappointment, and causes a renewal of excitement which it may require weeks to subdue. In other states of a case, the visits of friends may not only be unobjectionable, but useful, and to those by whom this is regulated it must always be more pleasant to solicit than to decline them.

"The visits of strangers among the patients are often much less objectionable than those of friends and relatives, but even these, if not properly regulated, may produce bad effects. A larger company than usual passing through the wards at one time, rarely fails to produce among the insane a degree of excitement not generally observed; and if to this happen to be added anything like frivolity of behaviour, or thoughtlessness of conversation, the effect is noticed for some hours afterwards."—*Second Report*, pp. 26, 27.

Deception should never be practised on the insane. It is almost invariably followed by bad results. Vulgar, illiterate, and inexperienced persons are disposed to invest the insane with a feeling of superstition and horror. More than half of these horrors will be destroyed, and the chances of recovery increased, whenever the whole community can look upon the insane as upon other invalids, suffering under a disease as curable in the early stages as many others;—and can believe that, when restored, an individual who has been thus afflicted is as worthy of confidence and respect, and as capable of resuming his position in the world, as though he had recovered from a fever or other affection, in which the manifestations of his mind had been temporarily deranged. Patients can then be made to understand, that a hospital is only a place prepared by enlightened benevolence for the treatment of these affections, requiring, as they do, a greater diversity of means, and more varied and expensive arrangements, than are available in the ordinary hospitals, or at their own homes.

Under these circumstances, some patients, if told candidly why they were removed from home, and where they were going, would acquiesce in the arrangement with cheerfulness; and if persuasion should fail, it would still, in nearly every case, be better to use sufficient force to effect the object, than to lose the respect and confidence of the patient by employing deception.

Many sensitive individuals find it exceedingly difficult to forgive the deception by which they have been brought from home; they brood over it for months,—consider it a proof that their friends are not capable of appreciating the true state of their minds, and in some very intelligent cases, it has really appeared to be a source of greater grief than all the privations attending their residence in a hospital.

We are glad to hear the medical writer of the Report observe, that—

"The free and systematic exercise of the muscles of the body, in the open air, is unquestionably one of the most powerful means of overcoming that nervous irritability which we constantly find among our patients,—of breaking up a train of morbid thought, or preventing the indulgence of vicious habits; all of which are often to be traced directly to sedentary pursuits in impure air, and an exercise of the mind totally disproportioned to that of the body. By these, a state of the system is often engendered, that no course of medication, without a total change of habits, can remove."

After detailing at some length the system of moral treatment pursued in the asylum, which appears to be based upon the most enlarged, liberal, and humane views of the disorders of the mind, the following observations are made on the subject of—

*Restraint and Seclusion in the Treatment of the Insane:*—"It is not to be concealed, that we always have in our family some with that unfortunate temperament that blackens the fairest scenes,—distorts the purest motives, and misconstrues the kindest actions; and that many require some more decided restraint, until the violence of their attack has subsided.

"No hospital for the insane can ever be without restraint;—the very character of the building—the laws for its government, and the supervision and discipline that are required, impose a wholesome restraint upon all who enter its walls. Fortunately, the discipline and restraint which the necessity of the case demands, can hardly prove injurious. The same cannot be said of the means formerly believed necessary, the evils of which were of so terrible and lasting a character, that too much pains cannot be taken to diffuse more correct and enlightened views in regard to it.

"*Seclusion* to guarded chambers, for a limited period, is of vast importance in the treatment of insanity, but, to prevent abuse, its duration must be under the immediate direction of a superior officer of the house. To no other persons can it be safely intrusted.

"Every year brings us cases to prove the danger of seclusion being improperly continued; and the bad habits which we have found most difficulty in subduing, have been traced directly to this cause; often combined, it is true, with the constant employment of some kind of apparatus, which effectually prevented the patient from taking proper care of his person, had he been so disposed.

"Patients steadily confined to their rooms are generally more addicted to the destruction of clothing and furniture—to filthy habits—and often offer greater violence to those about them, than when they have more freedom in their movements.

"Seclusion, for very short periods, I have found sufficient restraint for nearly every case under care during the past year, and with an average population of one hundred and fourteen, there have rarely been more than four or five confined to their chambers. On more than one occasion, for two or three weeks together, not a single male was thus restrained. At the time of writing this report, and during several previous weeks, there has been but one of each sex in this situation. If proper provision is made for seclusion, classification, and attendance, all the common kinds of restraining apparatus may be dispensed with in the treatment of insanity; but of the propriety of doing so, under all circumstances, I still entertain doubts.

"The error of dispensing with all apparatus in every case,—if error it is,—is, fortunately, one that will cure itself, and one not likely to be adopted by any person who is not actuated by pure motives and genuine philanthropy.

"Our invariable rule is to remove all restraint from the person of every patient upon his entering the hospital, and it is with extreme reluctance that it is ever re-applied.

"The completion of the lodges has contributed to diminish the already very small amount of restraining apparatus used in this institution. They were constructed with the express view of preventing even seclusion, by a strict classification of the patients in the halls; and on that account the rooms are only intended for night, or for the temporary confinement of very violent patients by day. The effects of these arrangements have been very striking. By proper association and strict supervision, very little glass has been broken, (although much is exposed,) and many patients have been prevented from tearing their clothes, until the habit seemed to have been entirely forgotten.

"It may be assumed, as the result of experience, that a diminution of restraint, with proper attendance, promotes cleanly habits and lessens noise, breakage, and tearing.

"Among the patients received directly into these lodges, were several persons whose hands had been constantly in muffs, or analogous kinds of restraint, for years before they entered this hospital. Immediately on their reception all restraint was removed, and in no one instance has it been re-applied.

"In each of these buildings are generally sixteen or seventeen patients. During the year, no apparatus for restraint has been applied, except in two cases, and it is rare that more than one patient of each sex are confined to their rooms. In the lodge occupied by the females no restraining apparatus has been employed.

"Had I felt anxious to make such a declaration, it would have been in my power to have stated, that during the past year, no restraining apparatus of any kind had been upon the person of a single patient of this hospital;—but believing, as I do, that its occasional employment may be conferring a favour on the patient, it has always been resorted to where there existed a proper indication for its use. The only indication for its use that is recognised in this hospital, is the positive benefit or safety of the patient,—never the trouble of those to whose care he is intrusted,—and the direct order of the physician or his assistant, the only authority under which it can be applied. The use of restraining apparatus ought rarely to be intrusted to other hands. Until attendants have learned by experience that ultimately they gain by avoiding its use—they rarely fail to suggest its employment, under improper circumstances—upon every occasion, indeed, when difficulty or danger is apprehended; instead of showing their own tact, by a resort to other expedients for controlling the patient.

"It has been truly said, that 'any contrivance which diminishes the necessity for vigilance, must prove hurtful to the discipline' of an hospital for the insane; and this is a strong argument against the ingenious contrivances that have been devised for this very purpose.

"Since my last report, one female patient was kept upon her bed for a few nights, by a very efficient and comfortable apparatus of leather. Wristbands, secured by a belt around the body, were used with four males, and mittens (all of leather) were kept upon the hands of three other men during a few days. A few hours were generally sufficient for all purposes. These were used when the disposition to injure themselves or others was particularly striking, or to prevent the indulgence of vicious habits."—*Second Report*, pp. 38—42.

One of the most difficult features in connexion with the management of asylums, is that relating to the character of attendants upon the insane. It is certainly most desirable that they should be possessed of a high moral character, a good education, strict temperance, kind and respectful manners, a cheerful and forbearing temper, with calmness under every irritation; industry, zeal, and watchfulness in the discharge of duty; and, above all, that sympathy with those under their care which springs from the heart, are among the qualities which are desirable, and as many as possible of which should be combined in those who are placed in this station.

When all these are found in one individual, and he has been instructed in the proper mode of performing his duties, his services to any institution and to the sick are truly invaluable. Such an attendant is really a benefactor to his species.

With a view of relieving the attendants from the wear and tear of body and mind, almost inseparable from their anxious and responsible vocation, Dr. Kirkbride, the physician to this asylum says he has been in the habit of giving as much variety as possible to the occupation, by combining out-door exercise with ward-duties, and affording a certain amount of time for entire relaxation, or for innocent recreation. This effectually produces good health, and the cheerfulness and equanimity of temper springing from it, which are so important in their intercourse with the insane. The patients are as much benefited by this course as the attendants themselves.

The subjoined observations are so replete with good sense, and calculated to dispel so large an amount of error, that we do not hesitate in transcribing them (without abridgment) to our pages. They refer to the subject of—

"*Popular Errors respecting Insanity.*—The condition of the insane is not influenced alone by the treatment they receive at home and in hospitals; their comfort and happiness is also, in many cases, deeply dependent upon a correct public opinion, and a proper appreciation by the community of the true nature of their malady.

"The insane complain, with cause, that their disease is not regarded as others are; that its character is misunderstood; and that, although the ridiculous ideas prevalent half-a-century ago are mostly exploded, some hardly less rational are still entertained by many persons of character and standing in the community.

"Insanity should be classed with other diseases. Many persons of fine feelings are extremely sensitive on this subject; they suffer deeply, and have their convalescence protracted, by a belief that there are numbers yet to be found who regard their disease as a reproach, and something to be remembered in all their future intercourse with them.

"It should never be forgotten, that every individual who has a brain is liable to insanity, precisely as every one who has lungs is liable to pneumonia, or, as every one with a stomach runs the risk at some period of being a martyr to dyspepsia. Prudence and a good constitution will often ward off complaints through a long life, but how often, even with the most careful, does disease commit fearful ravages. It is with insanity as with other affections. Our records, and those of other institutions, establish the fact, that scarce any age is positively exempt; that there is no profession, trade, or calling, but has representatives among its victims; that it is constantly met with where no hereditary taint can be discovered, and that in a great number of cases, no satisfactory cause for the disease can be assigned.

"As tending, in some measure, to give insanity its proper position among diseases, and confirming the impression, which it is always important to keep prominent before a patient, that it is curable, I have always preferred the name of Hospital, for institutions for its treatment, to all others that have been suggested; and it is very doubtful, if advantage has ever been derived from calling insanity by any other than its proper name.

"It has been too much the custom to say, without any qualification, that 'insanity is the greatest affliction that can befal humanity;' and many patients have had their wretchedness vastly increased by this common assertion. When neglected or aggravated by brutal treatment, it unquestionably becomes so. The proposition just referred to has originated from taking, as a type of the disease, some incurable case, labouring under the most violent and repulsive symptoms, and made hopeless, perhaps, by want of proper care, or by a course of management tending only to prevent recovery.

"Such cases do ultimately reach nearly every Hospital, but do not present a fair specimen of the disease, nor is it just that what may be perfectly true of them should be applied to all. Although it must ever be painful to look upon the wreck of a strong mind, it may be doubted whether, even among the incurable, there are not numbers who are comparatively happy, and have many enjoyments, if the proper feeling actuates those under whose care they live.

"The melancholy state to which these incurable cases are often reduced, and the striking contrast between them and those who have been restored, offer strong reasons for the general diffusion of correct views on this whole subject, for strenuous efforts for the establishment and liberal endowment of Hospitals enough to enable all in the early stages of the malady to partake of their advantages, and for leaving nothing undone, to have in and about those already in operation everything that can contribute in any way to promote the objects for which they have been erected.

"In a comparison of insanity with other diseases, it must be borne in mind that it presents the greatest diversity of aspect, and that the symptoms are in almost endless variety; that many cases are attended with very little suffering, require but little restraint of any kind, are not disabled from appreciating books or the society around them, or from enjoying many intellectual and physical comforts.

"It is to be remembered, too, that the result of experience is, that at least eighty per cent. of all cases, promptly and judiciously treated, recover, in periods varying from less than three months to one year, and upwards; that most of these continue in the enjoyment of perfect mental health, and are as competent to fulfil all their social and public duties as they were before the accession of the disease.

"It is not necessary to refer to distinguished names, whose history is familiar to most readers, and whose writings and labours after recovery have shown no impairment of their mental powers; for every physician who has had charge of many patients of this description will recal to mind examples of individuals occupying prominent public stations, members of all the learned professions, merchants, and indeed persons in every station of life, who, after their restoration, have returned to their former pursuits, and have shown, by their successful prosecution of them, that neither their mental powers nor their usefulness to society had been abridged by the malady under which they had been labouring.

"It may well be asked, whether other maladies may not sometimes be as much to be dreaded as this; whether certain forms of consumption, whether some varieties of cancer and various other malignant diseases, which, through continued suffering, lead on to certain death,—whether even blindness, total and irremediable, protracted during a whole life, may not be as terrible as the sorrows of insanity can be, when existing only for a limited period.

"Like other diseases, insanity has its peculiarities, of which one of the most trying to attached relatives and friends is, that generally the patient must leave home, and the management of the case be intrusted to strangers, and that the resources of a hospital are often required to give the best chance for a restoration.

"Many persons in the interior know nothing of Hospitals, and descriptions drawn solely from the imagination have often distressed patients and their friends, and have deprived them of advantages which they might have enjoyed. A removal from home may be necessary for other diseases; for the benefit of climate, it is a common prescription; and if in these cases health was to be obtained by entering a hospital, few would be found to object to such a course. Nor would the insane or their friends object, did they know that a hospital was only an establishment for the cure of disease; prepared and endowed by enlightened benevolence, provided with all the conveniences and fixtures likely to contribute to the restoration and comfort of its patients,—many of which are of too expensive a character to be attainable by individual means, — where, by the architectural arrangements of the building, and the regulations of the wards, nearly all restraint is avoided; where the law of kindness is the governing one; where the sick have practised persons constantly about them—are carefully nursed and guarded from harm—

shielded from the gaze and remarks of idle curiosity—and where all their peculiarities, and all the ramblings of a disordered intellect, are, as far as possible, known only to those whose duty and wish it is to prevent all exposure.

"The association of insane persons in a hospital is occasionally objected to. The perfection of treating the disease would undoubtedly be, to surround each patient with a sufficient number of intelligent and well-educated sane persons, familiar with the whole subject. This, of course, is totally impracticable; and after careful observation on this point, I do not recollect ever having seen a patient, where there was a proper classification, materially injured by coming in contact with his fellow-sufferers. In many cases, the effect is negative, and sometimes disagreeable to a patient, without being at all injurious. With the mass of our patients it has been advantageous; in a few, very strikingly so.

"The more intelligent and sensitive class of insane patients frequently complain that they are annoyed, in various ways, both before and after recovery, by many well-meaning but indiscreet persons, in their intercourse with them, forgetting the scriptural injunction, 'That all things whatsoever ye would that men should do to you, do ye even so to them;'—and they ask that their natural peculiarities should not be more critically noticed by the community than are those of other men;—while, in common justice, they believe that they should be considered sane, and possessing the same rights and feelings as others, until they exhibit symptoms that would indicate mental derangement in those who had never been suspected of having the disease.

"We can all call to mind cases of peculiarity of conduct, obliquity of views on certain subjects, and various eccentricities, that in our intercourse with the world scarce excite a passing remark, but which, in a hospital, are liable to be considered as the remains of the disease. So statements of facts, not known or not understood by those who hear them, when made by patients, are often reported as a proof of continued insanity;—and it is a matter of common remark, that individuals are often seen to walk and converse with patients, without a suspicion of their being so, but who, when informed of it, immediately suppose they detect indications of something wrong, even when none can be discovered by those whose duty it is to watch for every symptom of the disease.

"Those who have themselves been insane may do much towards correcting all these popular errors, by their own conduct after recovery, and by conversing calmly upon the subject; avoiding alike that frivolity which makes a jest of what is serious, and that morbid sensitiveness which is shocked by the slightest allusion to it. Those who are interested in the management of institutions for the insane,—the members of the medical profession, and all humane persons familiar with the subject, can do still more, by allowing no suitable occasion to pass without controverting the wrong views which they will constantly hear advanced in their intercourse with society."—*Third Report*, pp. 28-34.

Dr. Kirkbride, in his Fourth Annual Report for 1844, again reverts to the important subject of restraint. He maintains it as his opinion, that from information collected from nearly all the regularly organized institutions for the insane in his country, he has no doubt but that less real restraint is employed in them, at this time, than in the same number of similar establishments in any part of the world.

In America, cruelty, immoderate restraint, and ingenious but barbarous contrivances to control the insane, are rarely, if ever, found in properly-organized hospitals. When they do exist, it is in the almshouses, and jails, and in the private dwellings of the inhabitants.

The disuse of restraint, or the non-restraint system, as it has been called, means the disuse of mechanical means, for which, of course, other less objectionable forms must be substituted. A properly-constructed building, an efficient organization,—a more numerous body of more active and intelligent attendants,—all the means to prevent excitement,—all the power which sympathizing kindness and tact can bring to control it when produced, and temporary seclusion, when other means fail, are only a part of the varied modes by which mechanical means of restraint are rendered unnecessary.

Few intelligent men, familiar with insanity, could now be found to assert that restraining apparatus was frequently required, or that many patients could be benefited by its use. The question in controversy appears to be, whether its use is ever justifiable under *any* circumstances, or whether it can ever be employed without injury to the patient?

Believing firmly that the improper use of restraining apparatus, combined with long-continued seclusion, has been, and ever will be, productive of the worst effects, and go far to render intractable, curable cases of disease, Dr. Kirkbride is still of opinion, that a few of the simpler forms of mechanical means may occasionally be employed, with advantage to a patient. Judging from his experience, the per-centage in any hospital for whom these means are indicated is exceedingly small, and for considerable intervals none will be required. The rule should be that no restraint is to be employed, excepting under special circumstances; while at the same time it should be understood, that long periods of seclusion are not to take its place, as the effects of the latter might be still more injurious than the former.

There is one advantage resulting from the nearly entire disuse of restraining apparatus that should never be overlooked,—it brings into activity a constant supervision,—a genuine watchfulness, develops the capabilities of the attendants,—shows the great power of kindness and firmness, and brings out a species of tact, which would have been dormant, had mechanical means and seclusion been resorted to, to overcome violence, remove excitement, or cure bad habits.

After a careful review of the 592 cases, which have been under care since the opening of this hospital, Dr. Kirkbride says he has no hesitation in saying, that all might have been treated without the use of any mechanical means of restraint, had they started with such a determination, or had they felt any pride in making such a declaration. He feels equally confident, however, that in the few cases in which the mild means already referred to have been employed, the effect in some has been to prolong, if not preserve life,—in others, to diminish serious danger, and to shorten extreme violence, and in scarce a single one to have been productive of the slightest injury of any kind.

In the Pennsylvania Hospital the restraining apparatus is kept in the office of the physician, and, like medicine, is never prescribed, unless there is believed to be a decided indication for its use. It is never applied but by the express direction of the physician, and, when applied, never kept on longer than he believes it likely to obviate a great danger, or to promote the comfort and benefit of the patient.

We are glad to perceive, by the Reports of the Asylum, that the education and instruction of the patients receive a proper degree of attention. The opinion is too prevalent, that the insane require for their comforts—their enjoyments—their reading—their accommodation of nearly every kind, something radically different from what would have been their choice when sane. Differences at times are really necessary, and discrimination is to be used; but it will ultimately be found, that the nearer the circumstances of each case will permit an approximation to what is required by, and due to, the sane, the more rational and successful will be our treatment of the insane. We are too apt to believe that what has not been done in the treatment of insanity cannot be done, and rest satisfied with things as they now exist. What appears right and proper should be tried,—if tried with care and prudence, little injury can result from the experiment: a short time will satisfy a careful and intelligent observer whether good is likely to come from it; and even if it proves a failure, some new views or facts can hardly fail to have been derived from the trial.

Many persons, forgetting the great variety there is in mental diseases, and that a mind deranged on one subject may be sound on all others, seem astonished at some of the means of treatment, and some of the forms of occupation and amusement, now to be found about most well-conducted hospitals. The questions constantly asked—whether the insane do really enjoy any of these means, whether they really understand what is being done, or whether they are able to appreciate anything of the higher order of physical or intellectual enjoyment—is a proof that the true condition of a majority of this class is not so well understood, as to give to those thus afflicted that thoroughly liberal and enlightened treatment which is due to such a malady, and necessary for its early and certain removal. Our experience proves that, during some period of their disease, a majority of patients are able to appreciate all the courtesies and comforts of life, and to participate in most of its enjoyments, occupations, and amusements, in a restricted way, with quite as much zest as a majority of the community of which they were recently members.

In the following observations relative to the *attendants upon the insane* we cordially agree:—

"Of the value and necessity for a numerous corps of good attendants in every hospital, there ought not at this day to be any difference of opinion; and every superintendent must feel it an important part of his duties to see that the best within his reach are regularly employed. No man could have too many good qualifications for such a station, and every day and hour would give him opportunities of using all his gifts, to the great benefit of those around him. It is a station in which natural feelings and human temper are often sorely tried. To have in each ward an individual thoroughly and practically familiar with the whole subject—courteous and refined in manner—Christianly patient and benevolent in character—able to act as the guide, and counsellor, and friend of all the patients in their varying conditions—to direct with judgment, and act with tact in every emergency, would be making a great stride towards perfection in our institutions. The nearer we approach this, the nearer right we shall be.

"Most individuals when they first engage in these stations are perfect novices, and we should expect too much to suppose that their previous pursuits would qualify them to perform at once all their duties in a very perfect manner. When we know how utterly erroneous are the views of many, otherwise well-informed men—how injudicious their treatment of the insane—can we be surprised that new attendants are often at a loss, and generally require considerable training before their services are very valuable?

"To remedy in some measure these difficulties, and to elevate the character of the attendants, I propose to give to those employed in this institution, as my other engagements will permit, a regular course of instruction on the nature of their duties, embracing some general views of the character and peculiarities of the diseases which affect our patients—the principles which should regulate their intercourse with them, and with each other—the proper mode of proceeding in difficult cases, and such other matters as would be likely to give them a just sense of the importance and responsibility of their calling. Some such knowledge ought really to be possessed by every one before he becomes an attendant; and, when all these things are properly appreciated, every applicant should be regularly examined on these subjects before he is employed.

"No duty is more pleasant than to be able to acknowledge, as I am, the value of the labours of supervisors and attendants in this institution. They always have the power of earning the confidence of the officers and the respect and gratitude of the patients, and in numerous instances have been most successful in doing so."—*Fifth Report*, pp. 38, 39.

From an examination of the records of almost any institution, it will be found that a large proportion of its permanent population, from the long duration of their diseases, present but a feeble prospect for their perfect restoration; and on this account it has frequently been suggested, that hospitals for incurable patients, more cheaply organized and less perfect in their character than those for curing insanity, should be provided in every community.

These propositions, there is reason to believe, have generally been made without sufficient reflection, and our own observations would lead to a directly opposite conclusion.

In the first place, it is not possible to say with accuracy who are curable. There is not a year passes but that we have the satisfaction of seeing some restored to all their wonted mental vigour, who had been looked upon as without hope; while others of the same class have experienced an improvement in their condition which enabled them to return to society, and occasionally to engage in useful occupation and to indulge in many rational sources of amusement. Even if there was no risk of destroying in these every chance of returning reason, by condemning them to the exclusive society of incurables, and to the limited means of comfort which would most likely be appropriated to them, there are still reasons enough, in reference to those who are never to be restored, to prevent, in my estimation, a division of hospitals into curable and incurable.

It is no small matter, if a disease of the mind cannot be completely removed, that the mind is kept as far as possible from the lowest forms of dementia. The moral means which are to aid in the restoration of the curable are often efficient in sustaining the mental powers of those who are really incurable. Every intelligent person knows the effect of associatio ı upon his own mental powers and activity; he feels in himself, and sees in others, that the society of the moral, the educated and refined, has a steadily elevating and improving influence, while the companionship of the profligate, the ignorant and the degraded, is equally sure to gradually bring down the brightest intellect and to obscure the finest talents. So when the mind is primarily diseased, and the symptoms are of the slighter kind, the steady, untiring and judicious sympathy and kindness of friends will often dissipate the cloud that was gathering, and which, under other circumstances, would ere long have left the unmistakable imprint of its fearful ravages. At a later period, when the sufferer has passed through the early stages of his malady, and become, perhaps, the permanent resident of a hospital for the insane, kindness and sympathy are still efficient, respectful and courteous treatment are still appreciated, exercise and labour still bring their best reward, judicious conversation, reading, or even hearing the reading, of good books, the attendance of lectures, riding and walking, visits to objects of interest, variety in daily pursuits, good food, well cooked and properly served, neatness of person and dress, everything, indeed, that keeps the mind as well as the body steadily and pleasantly employed, is still productive of beneficial results.

To witness a mind that seemed lost completely restored to health and usefulness, is no ordinary gratification; but to the philanthropist it is only less than this, to see minds that cannot be restored kept from losing all their powers, and the possessors of them from becoming the wretched objects, mentally and physically, to which long continued neglect and ill treatment can then so easily reduce them.

It has been objected, too, that the curable must be injured by the presence of those who are incurable. A judicious classification will take all force from this objection, and those who argue thus must forget, that among the incurables of a large hospital are frequently ladies and gentlemen, whose good qualities of head and heart, whose kind feelings and courteous manners, would make their presence desirable in almost any company; whose few delusions injure no one, and the loss of whose society would be removing much of the attractions of a ward for even the most recent and curable cases. There are others who, although they have no decidedly good qualities to recommend them, still seem to add to the variety which most are partial to; and even the very worst of the chronic cases are rarely for a long time in a condition that is so melancholy and revolting, as are some of the most recent and curable for short periods.

While life lasts, every case should be considered under treatment—if not to cure, at least to keep it from becoming worse. Those who are likely to spend their lives in these institutions have, perhaps, the strongest claim on their friends, and those intrusted with their care, for everything that can tend to promote their comfort and happiness, and to preserve their mental and physical health.

Every year presents us with cases that should teach us never to despair—at least to treat all as though there was still hope of returning health—to surround our oldest and worst cases with as many as possible of the influences that would be likely to impress favourably the most recent and hopeful patients, and as few as can be of those which might tend to retard their restoration. We shall then be sure that our treatment of all is of the soundest and most enlightened kind, and for our efforts we shall often have to reward us, not only partial amendment where least looked for, but the cheering spectacle of minds gradually emerging from the chaos in which they had been involved, perhaps for long years, and slowly but steadily regaining their original powers, their sympathies with their fellow beings, and enabling their possessors to resume the place long vacant in the family circle, and again to become useful members of society.

The experience of our hospitals, in reference to chronic as well as recent cases, is important to the friends of patients; they are more deeply interested than any other class, and without their co-operation little can be done by the officers of our institutions. A steady perseverance in efforts for the cure of a patient, even for very long periods, is often most unexpectedly rewarded, and can rarely give any cause for ultimate regret.

We beg to direct the attention of our readers to the following observations, in reference to a—

"*Provision for the Habitually Intemperate.*—This subject is one of deep interest to the philanthropist, and is constantly brought to the notice of those who have charge of hospitals for the insane, by the frequent applications for the admission of improper cases of this description, by the difficulty which frequently exists in determining whether the individual should be admitted or not, and the earnest appeals for advice in reference to this unfortunate class of persons.

"Although the frequency of intemperance as a direct cause of insanity, may occasionally have been over-rated, still the records of this and of most other institutions of a similar character, show conclusively that even in this way it is a prominent and fearful one, the diseases of no less than 66 out of 663 men who have been received here being clearly attributable to this cause alone. If we could record all the cases which were produced by it indirectly, by the ill health which it engenders, by the loss of property and of character, the family difficulties which it excites, the activity given by it to the worst passions and vices, the silent grief over long-cherished hopes, and the deep mortification of sensitive and refined minds which result from it; its effects on others besides the victim of the habit,—on parents, wives, children, and friends, the number to whom intemperance is assigned as a direct cause in our tables, would but feebly portray the proportion of those who indirectly, but not less certainly, owe to it their insanity, as well as their other sources of permanent sorrow and domestic wretchedness.

"Where real insanity is the result of intemperance, a hospital for the insane is unquestionably the proper place for the victim of this wide-spread vice, and when mania-à-potu—which ought never to be received into an institution for the insane,—terminates in insanity, as it occasionally does, the same distinction is then proper for the wretched sufferer, whose case is likely to be of long standing, and the recovery always doubtful.

"An uncontrollable fondness for, and indulgence in, ardent spirits or other stimulants, with the usual results of such a course, are occasionally only symptoms of insanity, coming on in the progress of the case, often in individuals of the most correct habits, who had never before manifested such a propensity, and disappearing as the other symptoms of insanity are removed. In these individuals, of course, this peculiarity offers no reason for interfering with the ordinary disposition of such cases.

"There are, however, other and quite numerous classes of habitual drinkers, who are not suitable subjects for a hospital for the insane, but for whom some special provision should be made on their own account, and still more for the sake of their families and friends, and for the peace and quiet of the community.

"One of these classes is composed of individuals whose intemperance leads to acts of outrage against society, and brings grief and terror into quiet families, with ruin to their worldly prospects, but who seem to care little for reformation, and for whose acts insanity cannot be pleaded as an excuse. The seclusion of these persons brings temporary improvement, but nothing more, and if allowed they would, for limited periods, be frequently found in our hospitals for the insane, for admission into which they clearly can have no just claim.

"The moral effect produced on other patients in the wards, by the presence of such individuals, is almost always unhappy; they cannot legally be detained but for a short period; they are commonly indignant at the restraints which are necessarily imposed on them, and when discharged, they return to their homes only to renew the same scenes of debauchery and outrage. Insane patients object most strongly and reasonably to such associates, and with great justice protest against disease being placed on a par with vice, and misfortune with wilful debasement; claiming, with truth, that although insanity is a heavy affliction, it brings with it no reproach, and its acts sully no one with dishonour.

"There is another class, however—much more numerous, too, than is generally supposed—for whom advice is frequently solicited, and whose cases, on many accounts, are possessed of great interest. They differ in nearly every respect from those previously referred to, except an uncontrollable fondness for stimulants, and a moral weakness, which prevents their resisting the slightest temptation.

"When not under the influence of the habit, they are fully sensible of its enormity, and of the results sure to follow from its continuance, are anxious to reform, and willing to submit to almost any privation to effect the object. These are frequently persons of high standing in the community, possessed of wealth and every worldly comfort that could be desired. No business or profession is exempt, not even ministers of the Gospel. From the histories given by patients or their friends, it is common to learn that the sufferer is a man of liberal education, ample wealth, surrounded by an affectionate and devoted family, happy in all his domestic relations, and respected in the community; himself a truly benevolent man, active in works of charity, and ever ready to assist the suffering, yet, with all this, an uncontrollable fondness for stimulation is destroying everything; domestic happiness is gone, worldly ruin is impending, impaired health begins to foreshadow the coming destruction of a good constitution, and the future is even darker than the present.

"Such cases do occasionally enter a hospital for the insane, and their good qualities of head and heart render their presence quite unobjectionable; but there is much in the discipline of such institutions not pleasant to them, and the society they there meet is apt to become tiresome, so that they are likely to leave before their reformation is complete.

"When the pecuniary means of patients are ample, detached cottages, or apartments, disconnected with the regular wards, may be advantageously used; but only a limited number could thus be provided for, and then not under all the circumstances most favourable for a thorough reform.

"A certificate that the applicant for admission 'is insane,' signed by a respectable graduate of medicine, is required here, and, it seems to me, should be everywhere, preparatory to the admission of a patient into a hospital for the insane. This one regulation, of itself, will exclude most of the 'habitually intemperate,' and although a few do enter as monomaniacs, the number is comparatively small. Commonly, the stay of these cannot be insisted on for a sufficiently long period, and reformation is rarely the result of the attempt.

"For all these different cases some provision should be made, a retreat provided, where those who are anxious to reform should be surrounded by every influence likely to second their good intentions, and where society would be protected from those, who, with little care for the result, are not only ruining themselves, but destroying every good prospect of their families. The detention should be legalized, and not terminated but upon a proper medical or judicial investigation, and not regulated in any respect by the wishes of the patient or his friends.

"Such an institution should be under the direction of a well-educated and judicious physician, who should treat his patients as labouring under disease; and with kindness and firmness, a combination of medical and moral means, there is little doubt but that many good citizens would be annually restored to society; and where permanent reformation was found to be impracticable, individuals would be kept from habitual debasement, their families saved from ruin, and society protected from violence and disorder. It is a field for labour worthy of the active benevolence of the age."

We should like to see this important subject taken up by judicious persons, and much of the fallacy, error, and prejudice in association with it dispelled. Alas! how many hundreds are annually sacrificed at the shrine of intemperance, whose lives might have been spared had efficient means been resorted to at an early period, before the habit became fixed and confirmed. That there is a disease of the mind manifested solely in an uncontrollable desire for stimulating drinks, we have not a doubt; the more we see of the insane, the stronger is this conviction fastened on our minds. There is ordinary intoxication, and this may, to a certain extent, become a *habit;* but there is, apart from this, a form of insanity, exhibiting itself almost exclusively in a morbid yearning for intoxicating drinks. We have often seen cases of the kind, and as often have lamented our inability seriously to grapple with them until the disease has extended into positive delirium, and it is only at this stage that the law allows us to interpose!

It appears, from the last Report of this asylum, that during the past year the total number of patients in the establishment was 408.

The following is an account of the number of patients discharged during the year 1849:—

| | | |
|---|---|---|
| Cured | 104 | —25 per cent. |
| Much improved | 6 | } —10 per cent. |
| Improved | 33 | } |
| Stationary | 25 | |
| Died | 19 | |
| Total | 187 | |

The subjoined tables will be read with interest:—

TABLE I.—*Showing the number and sex of the admissions and discharges, since the opening of the Hospital, and of those remaining at the end of* 1849.

| | Males. | Females. | Total. |
|---|---|---|---|
| Admissions . . . . . . | 889 | 710 | 1599 |
| Discharges or deaths . . . | 771 | 607 | 1378 |
| Remain . . . . . . . | 118 | 103 | 221 |

TABLE II.—*Showing the ages of* 1599 *patients at the time of their admission.*

| | M. | F. | T. | | M. | F. | T. |
|---|---|---|---|---|---|---|---|
| Under 10 years | 1 | — | 1 | Between 50 and 55 | 48 | 34 | 82 |
| Between 10 and 15 | 1 | 2 | 3 | — 55 and 60 | 25 | 26 | 51 |
| — 15 and 20 | 45 | 46 | 91 | — 60 and 65 | 28 | 17 | 45 |
| — 20 and 25 | 153 | 103 | 256 | — 65 and 70 | 10 | 12 | 22 |
| — 25 and 30 | 164 | 114 | 278 | — 70 and 75 | 10 | 17 | 27 |
| — 30 and 35 | 131 | 76 | 207 | — 75 and 80 | 4 | 5 | 9 |
| — 35 and 40 | 119 | 82 | 201 | — 80 and 85 | — | 1 | 1 |
| — 40 and 45 | 81 | 106 | 187 | | | | |
| — 45 and 50 | 69 | 69 | 138 | | 889 | 710 | 1599 |

TABLE III.—*Showing the number of single, married, widows, and widowers, in* 1599 *patients.*

| | Males. | Females. | Total. |
|---|---|---|---|
| Single . . . . . . . | 507 | 283 | 790 |
| Married . . . . . . . | 341 | 327 | 668 |
| Widows . . . . . . . | — | 100 | 100 |
| Widowers . . . . . . . | 41 | — | — |

TABLE IV.—*Showing the supposed cause of insanity in* 1599 *patients.*

| | M. | F. | T. | | M. | F. | T. |
|---|---|---|---|---|---|---|---|
| Ill health of various kinds | 136 | 117 | 253 | Nostalgia | — | 2 | 2 |
| Intemperance | 88 | 7 | 95 | Stock speculations | 2 | — | 2 |
| Loss of property | 51 | 21 | 72 | Want of employment | 22 | 2 | 24 |
| Dread of poverty | 2 | — | 2 | Mortified pride | 2 | 1 | 3 |
| Disappointed affections | 11 | 13 | 24 | Celibacy | 1 | — | 1 |
| Intense study | 15 | 4 | 19 | Anxiety for wealth | 1 | — | 1 |
| Domestic difficulties | 14 | 34 | 48 | Use of opium | 2 | 5 | 7 |
| Fright | 9 | 14 | 23 | Use of tobacco | 3 | — | 3 |
| Grief, loss of friends, &c. | 25 | 52 | 77 | Puerperal state | — | 53 | 53 |
| Intense application to business | 13 | — | 13 | Lactation, too long continued | — | 3 | 3 |
| Religious excitement | 33 | 28 | 61 | Uncontrolled passion | 4 | 6 | 10 |
| Political excitement | 3 | — | 3 | Tight lacing | — | 1 | 1 |
| Metaphysical speculations | 1 | — | 1 | Injuries of the head | 11 | 3 | 14 |
| Want of exercise | 4 | 1 | 5 | Masturbation | 11 | — | 11 |
| Engagement in a duel | 1 | — | 1 | Mental anxiety | 36 | 41 | 77 |
| Disappointed expectations | 4 | 4 | 8 | Exposure to cold | 2 | — | 2 |
| | | | | Exposure to direct rays of the sun | 15 | — | 15 |
| | | | | Exposure to intense heat | — | 1 | 1 |
| | | | | Unascertained | 367 | 297 | 664 |

TABLE V.—*Showing the ages at which insanity first appeared in* 1599 *patients.*

| | M. | F. | T. | | M. | F. | T. |
|---|---|---|---|---|---|---|---|
| Under 10 years | 2 | — | 2 | Between 50 and 55 | 26 | 21 | 47 |
| Between 10 and 15 | 14 | 9 | 23 | — 55 and 60 | 16 | 27 | 43 |
| — 15 and 20 | 87 | 75 | 162 | — 60 and 65 | 17 | 10 | 27 |
| — 20 and 25 | 193 | 134 | 327 | — 65 and 70 | 3 | 2 | 5 |
| — 25 and 30 | 169 | 140 | 309 | — 70 and 75 | 4 | 1 | 5 |
| — 30 and 35 | 125 | 86 | 211 | — 75 and 80 | 3 | 1 | 4 |
| — 35 and 40 | 102 | 66 | 168 | | | | |
| — 40 and 45 | 82 | 87 | 169 | | 889 | 710 | 1599 |
| — 45 and 50 | 46 | 51 | 97 | | | | |

TABLE VI.—*Showing the forms of disease for which* 1590 *patients were admitted.*

| | Males. | Females. | Total. |
|---|---|---|---|
| Mania . . . . . . . . | 429 | 373 | 802 |
| Melancholia . . . . . . | 181 | 181 | 362 |
| Monomania . . . . . . | 140 | 88 | 228 |
| Dementia . . . . . . . | 132 | 65 | 197 |
| Delirium . . . . . . . | 7 | 3 | 10 |

We cannot conclude our desultory review of these Reports, without expressing to Dr. Kirkbride, and to the other medical and general officers associated with him, the gratification we have derived from a perusal of this interesting history of their great national and benevolent institution for the care and cure of the insane. It gladdens our hearts to perceive how much our American brethren, connected with this department of medicine, are influenced by the highest, the purest, most benevolent and humane views of the treatment of those afflicted with this form of disease. May they go on and prosper, is our hearty and sincere benediction.

